# Патология теломер в онтогенезе у пациенток с синдромом Тернера

**DOI:** 10.14341/probl12869

**Published:** 2022-04-30

**Authors:** Р. К. Михеев, О. Р. Григорян, М. С. Панкратова, Е. Н. Андреева, Е. В. Шереметьева, Ю. С. Абсатарова, Н. Г. Мокрышева

**Affiliations:** Национальный медицинский исследовательский центр эндокринологии; Национальный медицинский исследовательский центр эндокринологии; Национальный медицинский исследовательский центр эндокринологии; Национальный медицинский исследовательский центр эндокринологии; Московский государственный медико-стоматологический университет имени А.И. Евдокимова; Национальный медицинский исследовательский центр эндокринологии; Национальный медицинский исследовательский центр эндокринологии; Национальный медицинский исследовательский центр эндокринологии

**Keywords:** синдром Тернера, синдром Ульриха–Тернера, теломеры, моносомия, бесплодие, хромосомы, мозаицизм

## Abstract

В медицинской литературе отмечаются попытки проведения исследований in vivo c целью подтвердить/опровергнуть ускоренное укорочение теломер у лиц с синдромом Тернера по сравнению с обладателями физиологически нормального кариотипа. Несмотря на весь накопленный клинический опыт ведения пациентов с синдромом Тернера, ключевые омиксные (протеомные и метаболомные) аспекты заболевания остаются «белым пятном». Основными их недостатками являются малый объем и избегание математической оценки корреляции с соматическими заболеваниями (ишемическая болезнь сердца, гипертоническая болезнь, пороки развития сердечно-сосудистой системы). В связи с чем организация многолетних международных мультицентровых исследований in vivo с привлечением клиницистов и молекулярных биологов и набор обширной выборки пациентов, в т.ч. с «мозаичным» кариотипом, в современной науке являются актуальными.

## Abstract (en)

According to present medical databases there many trials to provide in vivo researches in vivo to confirm/refute shortening process of telomeres among patients with Turner syndrome. Despite the successful clinical experience of providing such patients with Turner syndrome, a lot of omics (proteomic and metabolomic) aspects still stay unclear. Main disadvantages of most researches are small volume and minimized mathematical correlation with chronic disease (coronary heart disease, essential hypertension, cardiovascular malformations). Finally, organization of international prospective multi-centered researches in vivo including patients with mosaic cariotype and co-operation between physicians and biologists are optimal solutions for this present problem.

В начале ХХ в. отечественная медицина пережила экзистенциальный экзамен на выживаемость: несмотря на катастрофические последствия Первой мировой и Гражданской войн, величайшими клиницистами и теоретиками был достигнут прорыв в появлении и развитии новых разделов клинической медицины. Одним из примеров стала организационная деятельность основателя отечественной эндокринологической школы профессора Н.А. Шерешевского (1850–1941). В 1925 г. Н.А. Шерешевский описал 25-летнюю пациентку с необычным фенотипом: длина тела 132 см, отсутствие вторичных половых признаков, короткая шея с крыловидными складками, низкий рост волос, микрогнатия, высокое небо, широко расставленные соски [1]. В 1938 г. эстафету советского ученого «принял» H. Turner, дополнивший симптомокомплекс такими признаками, как вальгусная деформация локтевых суставов и дисгенезия гонад (яичников). Описанный комплекс симптомов на территории бывшего СССР получил название «синдром Шерешевского–Тернера», в свою очередь, в англоязычной литературе — «синдром Тернера» (англ. «Turner Syndrom»), в немецкоязычной — «синдром Ульриха–Тернера » (нем. «Ullrich–Turner Syndrom») [2].

В 1959 г. знаковым событием стали исследования британского цитогенетика Ch.E. Ford, определившие геномную первопричину данной аномалии — моносомию по Х-хромосоме [1]. По результатам дальнейших генетических исследований, цитогенетические критерии для синдрома Тернера значительно расширились с учетом мозаицизма: в медицинской литературе описано не менее 60 фенотипических вариантов, обусловленных разнообразием лежащих в основе цитогенетических паттернов (45X0 — классический вариант; 46,X,i(Xq) + мозаики с линиями клеток i(Xq); 46,X,del (Xq) + мозаики с линиям клеток del(Xq); 45X0/46XY; 45X/46XX) [3]. Одним из наиболее характерных признаков для большей части фенотипов является первичное бесплодие, обусловленное развитием первичной недостаточности яичников. В 1966 г. при аутопсийном исследовании абортивного материала зародышей с анэуплоидией Singh и Carr. (1966) была обнаружена сохранность зародышевой ткани яичников в течение I триместра беременности с последующим замещением соединительной тканью [4]; в 2003–2004 гг. Modi, Sane и Bhartiya методом обнаружения апоптотической фрагментации ДНК (TUNEL assay) определили сроки апоптоза зародышевых клеток при синдроме Тернера — с 15-й по 22-ю неделю беременности [5].

В 2006 г. в процессе определения первопричины первичной яичниковой недостаточности зародышевых клеток Keefe, Marquard и Liu выдвинули оригинальную гипотезу о возможной взаимосвязи апоптоза зародышевых клеток и укорочении длины концевых участков хромосом — теломер [6].

Теломеры (термин предложен в 1932 г. американским биологом Германом Джозефом Меллером (1890–1967) представляют собой защитные нуклеопротеидные структуры, выполняющие функцию стабилизации и защиты генетического материала от воздействия внешних неблагоприятных факторов. Длина теломеры составляет в среднем 10–15 килобаз (1 килобаза — 1000 азотистых оснований); данные структуры состоят из множества гексамерных нуклеотидных повторов (ТТАГГГ), локализованных на 3’-конце ДНК и сопряженных с 6 видами белковых комплексов «шелтеринов» (TRF1, TRF2, TIN2, RAP1, TPP1, POT1) [7][8]. При репарации ДНК, обусловленной необходимостью поддержания генетического гомеостаза, отмечается укорочение теломер (в среднем на 50–200 нуклеотидных повторов) и в конечном счёте апоптоз, что дает основания описывать теломеры как «своеобразные биологические часы». Соответственно, усугубляющаяся со временем дисфункция теломер влечет за собой фатальные последствия для клеток и для организма в целом: мутации, аберрации, активация протоонкогенов с последующим опухолевым ростом.

В связи с тенденцией к персонализации медицины и поиску геномных, протеомных и метаболомных предикторов внутренних болезней активно изучается корреляция длины теломер у пациентов с соматической патологией: нарушениями углеводного обмена [9][10], сердечно-сосудистыми заболеваниями [11], воспалительными заболеваниями желудочно-кишечного тракта [12], ожирением [13–15] и т.д.

Универсальным показателем реализации биологической программы организма является его воспроизводство: в отличие от мужчин, период половой активности которых может длиться до глубокой старости, у женщин данный период ограничен периодом жизни между менархе и снижением овариального резерва с переходом в менопаузу. На основании такой догмы Keefe et al. (2006), Kalmbach et al. (2013) независимо друг от друга выдвинули «теломеразную гипотезу» о физиологической сопутствующей связи между убыванием длины теломеров и объемом овариального резерва в основе формирования первичной яичниковой недостаточности у пациенток с синдромом Тернера [6][16]. В частности, обсуждался вопрос о заведомо запрограммированном ускоренном укорочении теломер в эмбриональном периоде [17]. Авторы гипотезы предполагают наличие нарушения формирования веретена клеточного деления у организмов с моносомией X0 с участием укороченных теломеров в периоды лептотены, зиготены и перехода к пахитене — ключевых стадий профазы I мейотического деления предшественников оогоний. При более запущенном укорочении длины теломер, обусловленном прямыми и косвенными межгенными взаимодействиями (в т.ч. и эпигенетическими), активируются сигнальные пути апоптоза и гибели гоноцитов еще на этапе эмбриогенеза.

В 2018 г. совместными усилиями исследователей из Института наследственной патологии (г. Львов, Украина) и Жешувского технологического университета была проведена экспериментальная работа, касавшаяся теории теломер-обусловленного происхождения геномных и хромосомных аберраций при различных формах анеуплоидий [18]. Генетический материал был выделен путем фенол-хлороформной экстракции, высаливания и растворения в трис-ацетатном буфере (pH 8,0) из ворсин хориона 105 образцов абортивного материала (в период между 5–12-й неделями гестации). Относительная длина теломер измерялась с применением полимеразно-цепной реакции в реальном времени (Real-time polymerase chain reaction). Результаты представлены в табл. 1.

**Table:** Таблица 1. Распределение совокупности абортивного материала по основным нозологическим единицам (Huleyuk et al., 2018)Table 1. Distribution of the population of abortive material by main nosological units (Huleyuk et al., 2018)

Механизм выкидыша	Синдром / Кариотип	Число образцов
Самопроизвольный	Физиологическая норма (46,XX/46,XY)	32
Синдром Кляйнфельтера (69,XXN)	13
Трисомия 16	13
Синдром Дауна (трисомия 21)	10
Артифициальный	Синдром Шерешевского-Тернера (45,X0)	12
Физиологическая норма (46,XX/46,XY)	25

По результатам оценки и сравнения относительной длины теломер было выявлено, что длина теломер в образцах с самопроизвольным выкидышем (нормальный кариотип, синдром Тернера, трисомия 21, трисомия 16, триплоидия) в целом достоверно меньше по сравнению с материалом, полученным в результате искусственного прерывания беременности. Данные результаты являются неспецифичными в отношении синдрома Тернера, т.к. демонстрируют разницу между совокупностью образцов с нормальным кариотипом (46,ХХ/46,XY), с одной стороны, и совокупностью образцов с разнообразными анеуплоидиями — с другой. В данном исследовании сравнительные исследования между подгруппами в рамках последней совокупности не проводились, что дает пищу для дальнейших изысканий в данном направлении с расширенной выборкой в будущем. Полученные результаты более подробно представлены ниже в табл. 2.

**Table:** Таблица 2. Относительная длина теломер в абортивном материале с различными формами анеуплоидии и нормальным кариотипом (Huleyuk et al., 2018)Table 2. Relative length of telomeres in abortive material with various forms of aneuploidy and normal karyotype (Huleyuk et al., 2018)

Исследуемая группа	Число образцов	Вариабельность	Средняя длина теломер (± SEM)
Моносомия (45,X0)	12	0,08-1,66, P>0,05	0,36
Трисомия 21	10	0,11-0,35, P>0,05	0,26
Трисомия 16	13	0,07-0,49, P>0,05	0,28
Триплоидия (69,XXN)	13	0,11-0,37, P>0,05	0,27
46,XX/46,XY(самопроизвольный выкидыш)	32	0,03-2,40, P=0,0015	0,64

В медицинской литературе отмечаются попытки проведения исследований in vivo c целью подтвердить/опровергнуть ускоренное укорочение теломер у лиц с синдромом Тернера по сравнению с обладателями физиологически нормального кариотипа. Так, в 2001 г. в European Journal of Human Genetics опубликовано проведенное на базе Университетской больницы г. Орхус (Дания) исследование, посвященное молекулярно-биологическим и геронтологическим аспектам синдромам Тернера. [19] В нем приняли участие с одной стороны — когорта из женщин молодого (23,7±1,9 года) и более старшего (47,1±3,8 года) возраста (n=30) с кариотипом 45X0 (экспериментальная группа), с другой — женщины молодого (24,8±3,0 года) и старшего (42,9±6,5 года) возраста (n=30) с кариотипом 46XX (контрольная группа). С помощью методики саузерн-блоттинга выполнена оценка скорости укорочения длины теломер (RTFL, Telomere Restriction Fragment Length), выделенных из культуры лейкоцитов периферической крови. В настоящем исследовании группой исследователей была выдвинута гипотеза о тенденции к более стремительному укорочению длины теломер у пациентов старшей возрастной группы с синдромом Тернера. Результаты наглядно отображены в табл. 3.

**Table:** Таблица 3. Корреляция длины фрагментов укорочения теломер в зависимости от возраста у пациенток с синдромом Шерешевского-Тернера и пациенток с нормальным кариотипомTable 3. Correlation of the length of telomere shortening fragments depending on age in patients with Shereshevsky-Turner syndrome and patients with a normal karyotype

Кариотип	Возраст, лет	Число пациентов	Длина фрагмента укорочения теломер, bp
45,XO	23,7±1,9	15	7011±52; P=0,3
46,XX	24,8±3,0	15	7285±917; P=0,3
45,XO	47,1±3,8	15	7357±573; P=0,6
46,XX	42,9±6,5	15	7221±621; P=0,6

Результаты исследований показывают, что убедительных данных за подтверждение выдвинутой гипотезы нет. Нельзя не отметить, что исследование было проведено в 2000– 2001 гг., задолго до разработки концепции «доказательной медицины» на относительно малой выборке (n=60), и не имело прецедента в научном мире, в связи с чем его повторное проведение по современным принципам является насущным.

По данным многолетних наблюдений, пациентки с синдромом Тернера имеют в онтогенезе особо высокий риск сердечно-сосудистой патологии, как врожденной, так и приобретенной. Согласно данным многолетнего (с 1977 по 2017 г.) клинического исследования на базе кардиохирургической клиники Mayo Clinic Rochester, из 281 пациентки выделена 51 пациентка (средний возраст — 28 лет, вариабельность от 8 до 41), прошедших по крайней мере 1 плановое кардиохирургическое вмешательство. По данным катамнеза, у пациенток с синдромом Тернера (45X0) обнаружен обширный спектр сочетанных терапевтических и кардиохирургических патологий (и их комбинаций), несвойственный индивидуумам с физиологически нормальным кариотипом 46,XX/XY (табл. 4). [20]. Наличие у пациентов генотипа 45,X0 (в т.ч. мозаичных вариантов) лежит в основе фенотипа особо высокого риска сердечнососудистой смертности, затрудняет на практике процессы предоперационной подготовки, выполнения высокотехнологичных кардиохирургических вмешательств, ведения послеоперационного периода, снижает шансы на здоровое долголетие.

**Table:** Таблица 4. Распределение пациенток с синдромом Шерешевского–Тернера, проходивших с 1977 по 2017 гг. стационарное лечение в Mayo Clinic Rochester по терапевтическим и кардиохирургическим нозологиям (Fuchs MM, Attenhofer Jost CH, et al., 2017)Table 4. Distribution of patients with Shereshevsky-Turner syndrome who underwent treatment from 1977 to 2017 inpatient treatment at the Mayo Clinic Rochester for therapeutic and cardiac surgical nosologies (Fuchs MM, Attenhofer Jost CH, et al., 2017)

Терапевтическая патология/состояние	Кардиохирургическая патология
Артериальная гипертензия — 26 (51%)	Двухстворчатый аортальный клапан — 33 (65%)
Гиперлипидемия — 21 (42%)	Коарктация аорты — 31 (61%)
Курение (в анамнезе) — 5 (10%)	Добавочная левая верхняя полая вена — 7 (14%)
Сахарный диабет — 9 (18%)	Аномалия легочных вен — 2 (4%)
Обструктивное апноэ сна — 7 (14%)	Дефект межпредсердной перегородки — 5 (10%)
Гипотиреоз — 29 (57%)	Дефект межжелудочковой перегородки — 4 (8%)
Фибрилляция предсердий — 7 (14%)	Открытый артериальный порок — 5 (10%)
Ишемическая болезнь сердца — 9 (18%)	Атриовентрикулярный септальный дефект — 1 (2%)
	Открытый коронарный синус — 1 (2%)

## ЗАКЛЮЧЕНИЕ

В распоряжении англоязычной текстовой базы данных медицинских и биологических публикаций Национального центра биотехнологической информации (NCBI) США имеется ограниченный объем исследований, посвященных патологии теломер при синдроме Тернера. Основными недостатками их проведения являются малый объем и избегание математической оценки корреляции с соматической патологией (ишемическая болезнь сердца, пороки развития сердечно-сосудистой системы). Несмотря на весь накопленный клинический опыт ведения пациентов с синдромом Тернера, ключевые омиксные (протеомные и метаболомные) аспекты заболевания остаются «белым пятном». Процесс прицельного изучения взаимосвязи аберрации теломер с пороками развития и функционирования жизненно важных органов и систем находится лишь на стадии инициации. Важнейшие условия для продвижения данного процесса — организация многолетних международных мультицентровых исследований in vivo с привлечением клиницистов и молекулярных биологов и набор обширной выборки пациентов, в т.ч. с «мозаичным» кариотипом.

## ДОПОЛНИТЕЛЬНАЯ ИНФОРМАЦИЯ

Источники финансирования. Исследование выполнено в рамках государственного задания «Влияние эпигенетических факторов на течение менопаузы у женщин с эндокринопатиями аутоиммунного генеза в рамках формирования модели “здорового старения”» 2021–2023 г.

Конфликт интересов. Авторы декларируют отсутствие явных и потенциальных конфликтов интересов, связанных с содержанием настоящей статьи.

Участие авторов. Мокрышева Н.Г., Григорян О.Р., Панкратова М.С., Михеев Р.К. — концепция и дизайн исследования; Михеев Р.К., Шереметьева Е.В., Абсатарова Ю.С. — сбор и обработка материала; Михеев Р.К., Григорян О.Р., Панкратова М.С. — написание текста; Григорян О.Р., Панкратова М.С., Андреева Е.Н., Шереметьева Е.В. — редактирование. Все авторы одобрили финальную версию статьи перед публикацией, выразили согласие нести ответственность за все аспекты работы, подразумевающую надлежащее изучение и решение вопросов, связанных с точностью или добросовестностью любой части работы.
